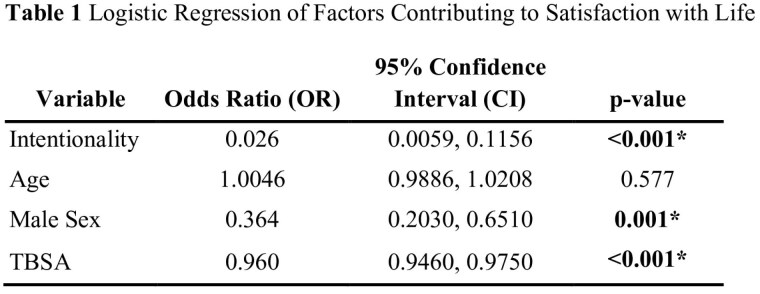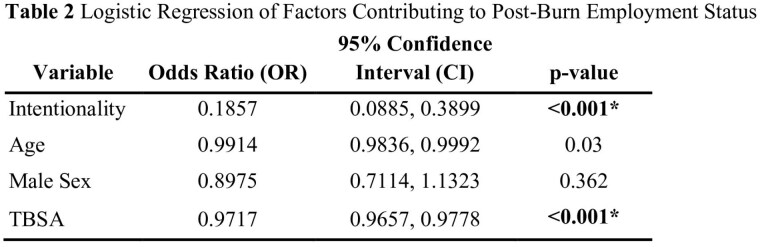# 118 Violent Burns Are Associated with Poorer Life Satisfaction and Unemployment After Injury: A Nationwide Analysis

**DOI:** 10.1093/jbcr/iraf019.118

**Published:** 2025-04-01

**Authors:** Artur Manasyan, Kara McMullen, Kimberly Roaten, Sunia Choudhury, Sarah Stoycos, Haig Yenikomshian, Maxwell Johnson

**Affiliations:** University of California Keck School of Medicine; University of Washington; University of Texas Southwestern; Harvard Medical School; University of California Keck School of Medicine; University of California Keck School of Medicine; University of California Keck School of Medicine

## Abstract

**Introduction:**

Assaults can result in violent burn injuries that have long-lasting effects on survivors. Resulting physical pain and psychological trauma can impact quality of life and employment. In this study, we aim to explore socioeconomic demographics and longitudinal psychosocial outcomes associated with violent burns.

**Methods:**

Adult participants admitted for acute burn injury between 1996 and 2023 within a multi-center longitudinal survey study were included in the present study. Violent burns were defined as circumstances of injury including suspected assault (both domestic and non-domestic) and arson. Patients with self-inflicted burn injuries were excluded. Participant demographics were summarized using descriptive statistics and compared between the violent and unintentional burn cohorts with Wilcoxon-Mann-Whitney and Chi-square tests. Mixed effects regression models were constructed to investigate longitudinal associations between violent burn injuries and anxiety, depression, posttraumatic stress symptoms, posttraumatic growth, and satisfaction with life. In each of these models, age, sex, total body surface area (TBSA) burn size and time were included as covariates. A logistic regression model examined associations between violent burns and employment status at 12 months post injury. This model controlled for pre-injury employment status, burn center, TBSA, age, and sex.

**Results:**

A total of 4,668 patients were included in the study, of whom 147 (3.15%) sustained violent burns. The violent burn cohort was more likely to be Black (p< 0.001), unemployed (p< 0.001), have lower income (p=0.001), and less likely to have completed graduate education (p< 0.001). Participants with violent burn injuries had larger burns (24.0 vs. 16.6% total body surface area, p< 0.001). Post-burn anxiety, depression, post-traumatic growth, and post-traumatic stress were not significantly associated with burn intentionality. However, survivors were more likely, after controlling for TBSA, to experience unemployment (p< 0.001) and lower satisfaction with life (p< 0.001) after injury.

**Conclusions:**

Violent burn injuries disproportionately impact historically marginalized and socioeconomically disadvantaged individuals. These injuries lead to poorer quality of life than unintentional burns, but there were similar impacts on psychological health regardless of intentionality of injury. Future research should focus on exploring dissatisfaction with life, barriers to employment, and the role of vocational counseling in addressing these challenges within this patient population.

**Applicability of Research to Practice:**

Our research highlights the need for targeted interventions and support systems to improve employment prospects and overall satisfaction for survivors. Implementing these findings in clinical practice can help tailor rehabilitation programs and social services to address the unique challenges faced by this population.

**Funding for the Study:**

The contents of this abstract were developed under a grant from the National Institute on Disability, Independent Living, and Rehabilitation Research (NIDILRR grant number 90DPBU0007). NIDILRR is a Center within the Administration for Community Living (ACL), Department of health and Human Services (HHS). The contents of this abstract do not necessarily represent the policy of NIDILRR, ACL, or HHS, and you should not assume endorsement by the Federal Government.